# Elevation of the Pelvis as a Means of Relieving Vomiting of Pregnancy

**Published:** 1891-08

**Authors:** James Grant

**Affiliations:** Consulting Physician General Hospital, Ottawa, etc.


### ELEVATION OF THE PELVIS AS A MEANS OF RELIEVING VOMITING OF PREGNANCY.

BY SIR JAMES GRANT, M. D.,
Consulting Physician General Hospital, Ottawa, etc.

In 1877 I was called to attend a lady in her first pregnancy, about the third month of utero-gestation. I learned that for fully ten days she had been unable to take food, and with great difficulty retained even the smallest quantity of liquid nourishment. Almost every form of treatment had been tried without any apparent good result. As a last expedient, I decided to test the effect of elevation of the pelvis, which was accomplished by lowering the head and thorax, and placing several pillows under the sacrum. In a short time the change for the better was encouraging, and continuing the position at intervals for a few hours, in two days the marked improvement in the system was quite evident, and utero-gestation proceeded to the full term without any return of this abnormal condition.

Within the past month, two cases of severe vomiting in early pregnancy came under observation, in both of which I adcpted the same treatment, with equally satisfactory results.

Guemot, referring to the rational treatment of vomiting during pregnancy, says that a morbid and abnormal state of the uterus, the nervous system, as the carrier of reflex action, and the stomach, are the prime factors in the malady. The idea of Smellie's, that the complaint is "chiefly occasioned by fulness of the vessels of the uterus," certainly is most rational. The elevation of the pelvis gradually lessons the quantity and force of the blood in the uterine vessels, and thus reduce the quasi-irritability, or, as Dr. James McGill terms it, "the irritability of the nerve elements" in the uterine nervous system, the abnormal influence of which, prior to the change of the pelvic position, had been rapidly telegraphed to the spinal and gastric nervous centres.

